# Continuous quality monitoring in the field: an evaluation of the performance of the Fio Deki Reader™ for rapid HIV testing in South Africa

**DOI:** 10.1186/s12879-020-4932-0

**Published:** 2020-05-04

**Authors:** Lara Noble, Lesley Scott, Lynsey Stewart-Isherwood, Seponono John Molifi, Ian Sanne, Pedro Da Silva, Wendy Stevens

**Affiliations:** 1 Department of Molecular Medicine and Haematology, School of Pathology, Faculty of Health Sciences, University of the Witwatersrand, Johannesburg, Gauteng South Africa; 2 National Priority Programme, National Health Laboratory Service, Johannesburg, Gauteng South Africa; 3 BroadReach Consulting, Johannesburg, Gauteng South Africa; 4 Strategic Evaluation Advisory and Development Consulting, Johannesburg, Gauteng South Africa; 5 Right to Care, Johannesburg, Gauteng South Africa

**Keywords:** Automated rapid strip reader, Continuous quality monitoring, HIV, Rapid diagnostic test, Deki reader

## Abstract

**Background:**

Rapid diagnostic tests (RDTs) are a cornerstone of HIV diagnosis and rely on good quality processing and interpretation, particularly in the era of test and treat. The Deki Reader (Fio Corporation®, Toronto, Ontario, Canada) is a portable device designed specifically for analysing RDTs and was selected for evaluation in South Africa in the context of HIV RDT analysis.

**Methods:**

This study consisted of a laboratory evaluation and two-part field evaluation of the Deki Reader v100, covering two RDT testing algorithms, and an evaluation of the continuous quality monitoring through the Fionet™ web portal. Based on user feedback from the field evaluation, the device underwent hardware and software redesign, and the Deki Reader v200 was evaluated in the laboratory. Ethics approval for this evaluation was obtained from the University of the Witwatersrand Human Research Ethics Committee: M150160.

**Results:**

The intra- and inter-device laboratory precision of the Deki Reader v100 were 98.3 and 99.2% respectively, and 99.3 and 100% for the Deki Reader v200. The laboratory concordances compared to standard-of-care reporting were 99.5 and 98.0% for the two respective models, while sensitivity and specificity were 99.5 and 99.4% for the Deki Reader V100 and 100 and 93.1% for the Deki Reader V200 respectively. Screening and confirmatory concordances in the field were 99.3 and 96.5% under algorithm 1 and 99.7 and 100% under algorithm 2. Sensitivity and specificity for the field evaluation were 99.8 and 97.7%. Overall robustness of the device was acceptable and continuous quality monitoring through Fionet™ was feasible.

**Conclusions:**

The Deki Reader provides an option for improved and reliable quality assessment for rapid diagnosis of HIV using RDTs to enhance the quality of healthcare at the point-of-care. However, the introduction of new RDTs and modification of current algorithms necessitates ongoing and agile RDT reader adjustments, which will require cost modelling to ensure sustainability of devices implemented into national HIV programs.

## Background

Rapid diagnostic tests (RDTs), with their simple strip-based technology, have been a cornerstone of disease diagnosis for decades. In many resource-limited settings, HIV diagnosis is now achieved using only RDTs, allowing for rapid scale-up of HIV testing services (HTS) [1], in an effort to meet the 90–90-90 [2] and ultimately 95–95-95 [3] goals. Closely linked to this are the game-changing studies showing the value of early antiretroviral therapy (ART) initiation [4–6], which ultimately led the World Health Organisation (WHO) to recommend a universal test and treat (UTT) program for HIV [7]. This initiative has been adopted by 93% of low- and middle-income countries and by 100% of the designated ‘fast-track’ countries [8], including South Africa [9]. Ongoing maintenance of the quality of rapid HIV diagnosis is critical and difficult to achieve at scale within current programs.

In addition to robust manufacturing, RDTs rely on good quality processing and interpretation. A recent systematic review [1] showed that suboptimal testing strategies, failure to follow algorithms, human error and inadequate supply chain management, amongst others, contribute to misdiagnosis globally. This analysis of 25 studies showed that users frequently perform or interpret the RDTs incorrectly, with some studies describing the use of expired RDTs [1]. Furthermore, RDT interpretation is subjective due to errors associated with visual interpretation [10, 11]. A South African evaluation by Strategic Evaluation, Advisory & Development (SEAD), a local non-governmental organisation, showed that test results were often read too early, with an overall process compliance if not immediately HIV positive of 3.4% nationally, with extreme non-compliance in KwaZulu Natal (9.7%) and Limpopo (8.6%) provinces [12], both of which have high prevalence of HIV. A recent audit of selected South African clinics showed that RDT testing practices were poor, with algorithm non-adherence, a lack of external quality control, and a lack of understanding when results were discordant, all of which may compromise the quality of the results [13].

The WHO recommends that all HIV testing sites participate in external quality assessment programmes, involving regular observation of RDT performance by trainers or supervisors, and evaluation of well-characterised HIV specimens [14, 15]. Dried tube specimens can be distributed for proficiency testing [16–18], but they may not reach all HST testers [19]. However, in a setting such as South Africa, with 270,000 (240,000–300,000) new HIV diagnoses in 2017 alone [20, 21], the HTS programme is massive and dispersed over a wide geographical area, making these forms of quality monitoring extremely difficult with poor overall impact. Given this information, it is clear that improved quality measures are required for HTS, particularly in remote areas where quality management of proficiency testing may be lacking or impractical to deliver. Jani and Peter (2013) described how connecting devices at point-of-care (POC) to the central laboratory could allow for better monitoring of results and quality [22]. An opinion piece by Wedderburn et al. (2015) discussed how the use of an electronic reader could improve the quality of RDT results, allow for capture of data for surveillance and possibly linkage to stock monitoring [23].

**Fig. 1:**
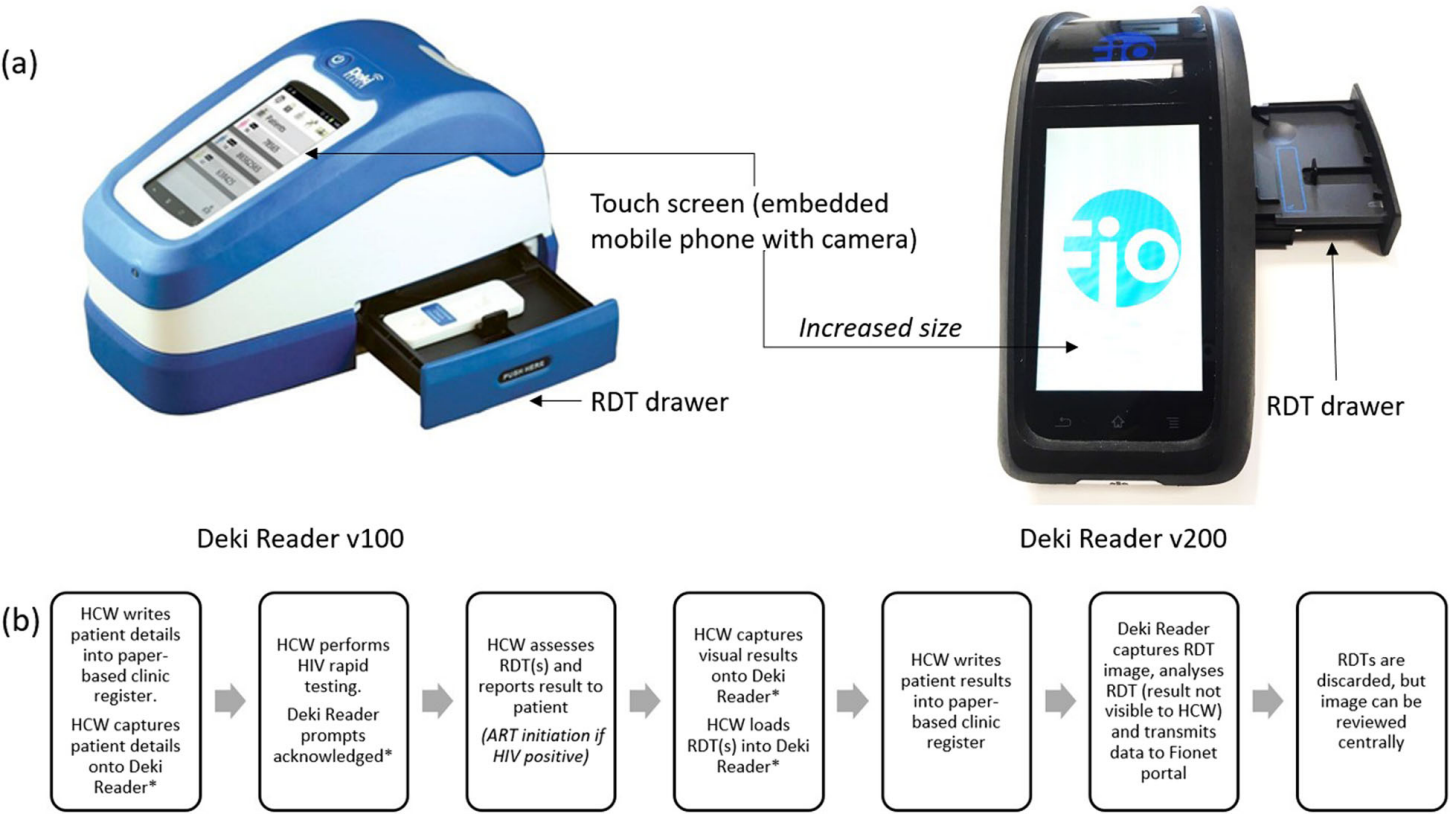
Deki Reader device and clinic work flow. **a** The Deki Reader v100 and the Deki Reader v200 are shown. Both have a small footprint, an adjustable drawer into which the RDT is placed and are based on an embedded mobile phone with a touch screen and a camera. Only the screen of the mobile phone is visible to the user and the Deki Reader v200 has a larger screen (shown). The battery bank and charger connections (not visible) are contained in the device housing. **b** Overview of clinic workflow for the Deki Reader Field evaluation. The Deki Reader processes were integrated into standard of care HIV testing, with extra steps performed by the HCW shown with an asterix (*). The HCW captures patient details onto the Deki Reader and follows the onscreen prompts to complete the RDT test; the prompts must be acknowledged. Once the appropriate RDT incubation time is completed, the HCW will visually interpret the result and capture it onto the Deki Reader, before inserting the RDT into the RDT drawer for automated analysis. The visual results are shared with the patient and HTS completed. Once the patient has exited the clinic, the HCW captures the results into the paper-based registers. The automated RDT results are automatically uploaded to the Fionet platform, enabling centralised result storage, but were not available to the HCW in this evaluation

There are a number of automated RDT analysers available, some linked to specific RDT brands and some with the potential to adapt to different RDTs. Applications created for smartphones are also available and are increasingly used by self-testers. One such device is the Deki Reader (Fio Corporation®, Toronto, Ontario, Canada), a portable device designed specifically for analysing RDTs and utilising smartphone technology (Fig. 1a). The agile software can be programmed for any number of RDTs and in-country diagnostic algorithms. The device can also be configured to provide step-wise guidance for RDT preparation and allows two-way communication between the tester and centralised assistance providers. Results, together with captured images of the RDT, are uploaded through a proprietary web portal, Fionet™, which allows data to be centralised and monitored on a continuous basis [24]. The Deki Reader has been evaluated for assessment of malaria RDTs and was shown to be comparable with visual RDT analysis [25–31], and the Fionet™ portal was recently shown to improve malaria diagnosis by enabling improved quality monitoring [24]. A recent study in Sierra Leone linking febrile illness to HIV used the Deki Reader to capture and upload the HIV RDT results [32]. However, no extensive evaluation of the Deki Reader for HIV RDT assessment in field settings (its intended use) is available. The authors hence performed a laboratory and field evaluation of the Deki Reader, including the use of the device as a tool for continuous quality monitoring, in Johannesburg (Gauteng province, South Africa).

## Methods

**Fig. 2:**
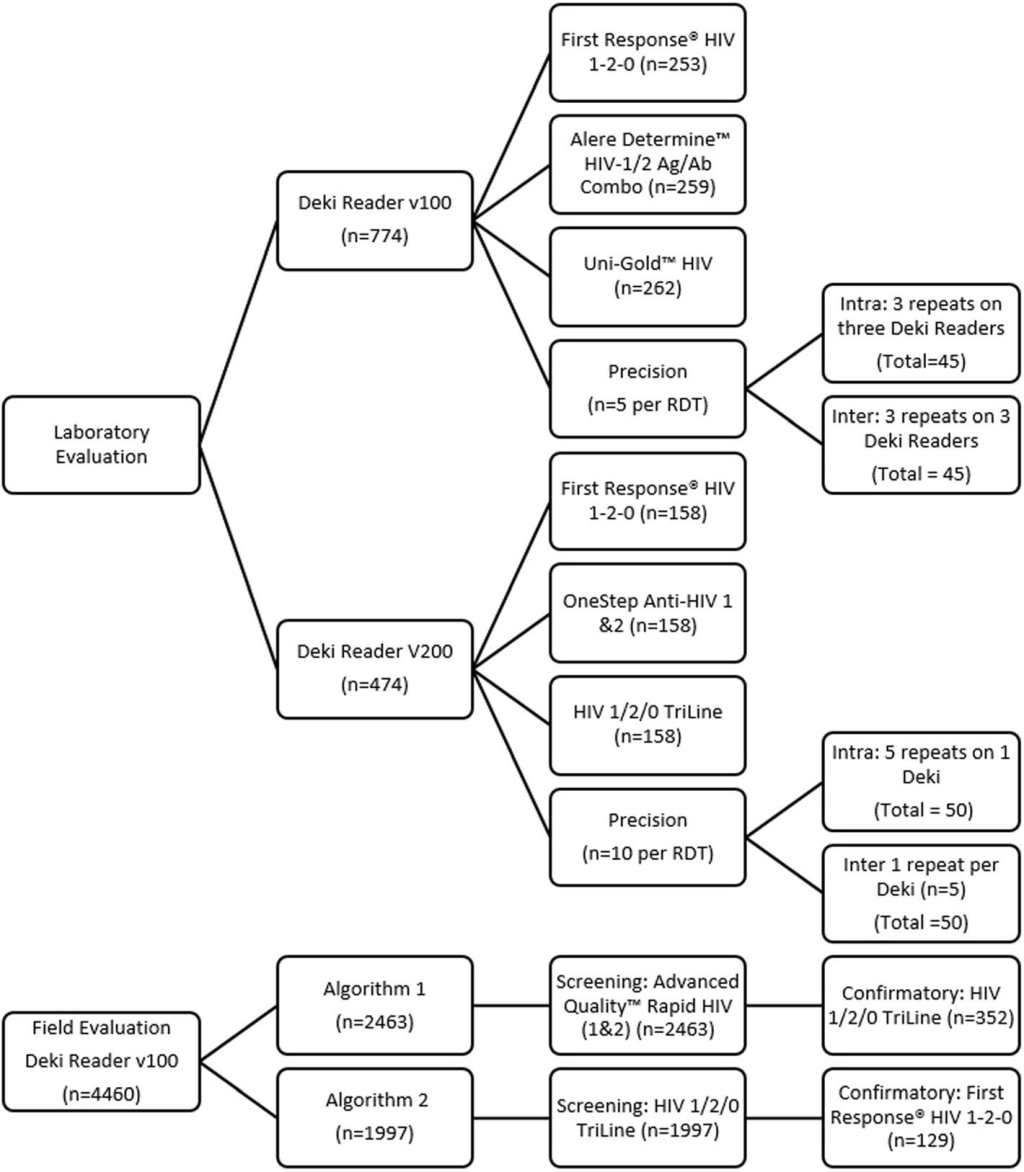
Summary of the Deki Reader Laboratory and Clinical Concordance Evaluations. Two separate laboratory evaluations were performed. The first (*n* = 774 specimens) evaluated the Deki Reader v100, while the second (*n* = 474 specimens) evaluated the upgraded technology, the Deki Reader v200. The devices were evaluated using different RDT brands that were selected based on changing country algorithms (planned and implemented). Each evaluation included concordance between automated and visual analysis, and a precision analysis. The phase 2 (clinical concordance) field evaluation was performed using two different RDT algorithms. The first field evaluation (*n* = 2463 specimens) used the Advanced Quality™ Rapid HIV (1&2) RDT for screening and the HIV 1/2/0 TriLine RDT for confirmation of positive results (algorithm 1). The second field evaluation (*n* = 1997 specimens) was performed after a national HIV RDT algorithm change, and used the HIV 1/2/0 TriLine RDT for screening and the First Response® HIV 1–2-0 for confirmation of positive results (algorithm 2)

This evaluation consisted of four parts, summarised in Fig. 2: (1) a laboratory evaluation and (2) a two-part field evaluation of the Deki Reader v100, (3) a second laboratory evaluation of an updated device (Deki Reader v200) and (4) an evaluation of the continuous quality monitoring through the Fionet™ web portal. The device underwent a physical redesign based on feedback from the first field evaluation, leading to the second laboratory evaluation to ensure continued accuracy and precision.

A target product profile (TPP) for automated RDT analysis devices was also designed. While this was not definitive, the Deki Reader was compared to this TPP to determine needs met within the testing environment. A critical future element for inclusion in the TPP is costing, which was beyond the scope of this evaluation.

### Ethics approval

Ethics approval for this evaluation (laboratory and field) was obtained from the University of the Witwatersrand (Johannesburg, South Africa) Human Research Ethics Committee (laboratory evaluation and field evaluation: M150160). All specimens used were considered to be residual specimens as no specimens were obtained specifically for the evaluation and results from the Deki reader were not used for patient management.

### Laboratory evaluation

All laboratory evaluations were performed in the Department of Molecular Medicine and Haematology, University of the Witwatersrand, Johannesburg, South Africa. Different RDTs were used in the two evaluations, based on changing national algorithms. RDTs used for the Deki Reader v100 evaluation (*n* = 774 specimens) were the First Response® HIV 1–2-0 (Premier Medical Corporation Limited, Daman, India; *n* = 253), the Determine™ HIV-1/2 Ag/Ab Combo (Alere Inc., Waltham, Massachusetts, USA; *n* = 259) or the Uni-Gold™ HIV (Trinity Biotech, Bray, County Wicklow, Ireland; *n* = 262). RDTs used for the Deki Reader v200 evaluation (*n* = 158 specimens) were the First Response® HIV 1–2-0 (Premier Medical Corporation Limited, Daman, India; *n* = 158), the HIV 1/2/0 TriLine (Abon™ BioPharm Co. Ltd., Hangzhou, China; *n* = 158)) and the OneStep Anti-HIV 1&2 (Guangzhou Wondfo Biotech Co. Ltd., Guangzhou, China; *n* = 158). Data was adjudicated by Fio as part of the software learning process and this data is presented.

For each evaluation, residual blood specimens with a known HIV status were obtained from the National Health Laboratory Service (NHLS) CD4 Flow Cytometry Laboratory (University of the Witwatersrand Medical School, Johannesburg, South Africa) and the NHLS Pathology Laboratory (Charlotte Maxeke Johannesburg Academic Hospital, Johannesburg, South Africa). RDTs were prepared according to manufacturer instructions and evaluated (read) by a laboratory professional. The visual (user or standard of care (SOC)) result was manually captured onto the Deki Reader and uploaded to Fionet™. The RDT was then inserted into the Deki Reader for automated analysis; this result was automatically uploaded to Fionet™. The two analyses were compared to assess the concordance between visual and automated interpretation, using the visual (SOC) result as the gold standard.

Further to this, precision analyses were performed using both HIV positive and HIV negative specimens. The Deki Reader v100 was tested using five different specimens per RDT (*n* = 3) and each RDT was read three times per each of three Deki Reader v100 instruments. The Deki Reader v200 was tested using ten different specimens that were tested on each RDT (*n* = 3), with each RDT being read five times on one instrument and once across five different Deki Reader v200 instruments. The latter protocol introduced more stringent precision analysis to assist the developer for improved software optimisation as a lesson learnt during the v100 evaluation.

### Field evaluation

The field evaluation was performed in collaboration with a local non-governmental organisation, Right to Care (Johannesburg, South Africa), between 5 May 2017 and 9 April 2018. Six key performance indicators were selected for this evaluation: (1) overall robustness of the Deki Reader, (2) efficient record upload speed, (3) connectivity needs and reliability, (4) quality data capturing concordance between visual and automated result interpretation, and (5) end-user (HCW) acceptance of the Deki Reader. HCWs and programme staff involved in the field evaluation attended a two-day training workshop, which included an introduction to the Deki Reader v100 hardware and software, and hands-on testing using the Deki Reader v100. Project managers received further training in the Fionet™ web portal software, learning how to remotely monitor and interact with the HCWs; filter data, reports and queries; and to manage quality related issues, including troubleshooting during RDT processing.

All patients attending the clinics for HIV testing services (HTS) were informed of the study and, whether or not they elected to enrol, were managed according the South Africa National HIV Guidelines [32] and the more recently adopted WHO ART guidelines [33]. HCW performed HTS as per SOC and residual RDTs were inserted into the Deki Reader v100 for testing, with results being available for neither the HCW nor the patient. The patient was required to wait for the HCW to enter their data onto the device and for the blank RDT to be captured before testing, as well as to wait for their test result (visual read), which also needed to be captured onto the Deki Reader immediately to adhere to test time parameters (Fig. 1b). These residual RDTs were assigned random study numbers by the HCW to ensure no linkage to patient data and visual results were captured onto the device. The RDTs were then inserted into the device and a high-resolution (5MP) image captured for automated analysis. All data were automatically uploaded to the Fionet™ portal.

The first part of the evaluation (**phase 1: 5 May to 5 October 2017**) assessed the suitability of the Deki Reader to withstand field conditions and the connectivity needs for reliable data transfer (indicators 1 to 3), as well as HCW acceptance (indicator 4). Three HTS clinics (Johannesburg Health District, Gauteng, South Africa) participated in this evaluation (*n* = 20 Deki Reader v100 devices) and 23 HCWs were trained. Themba Lethu Clinic (Helen Joseph Hospital, Johannesburg) is a fixed HTS clinic and had eight devices installed. The other participants were mobile HTS clinics servicing two different regions within the Johannesburg Health District and were managed by community teams; each were allocated six devices. The RDT testing workflow followed the algorithm stipulated in the HIV testing services policy (6), which was HIV screening using the Advanced Quality™ Rapid HIV (1&2) Test (InTec PRODUCTS, Inc., Xiamin, China) and HIV positivity confirmed using the ABON™ HIV 1/2/0 Tri-line Human Immunodeficiency Virus Rapid Test Device (Abon Biopharm (Hangzhou) Co., Ltd., Hangzhou, China). The uploaded images were used for device ‘training’ and automated reporting was not available for this phase. Information regarding the record upload speed (acceptability criteria > 90% in < 10 min), and robustness of the instrument in the field (system failures) was captured from the Fionet™ portal. All data was stored on the Fio Corporation server with restricted access.

The second part of the field evaluation (**phase 2: 8 October 2017 to 9 April 2018**) assessed the quality of the data recorded (acceptability criteria > 95% data fields captured by the HCW; 10% of records checked), including error rates and invalid results obtained, the concordance between the visual and automated RDT assessments, and the HCW acceptance of the Deki Reader v100 (indicators 4 to 6). Eight sites, with a total of 23 Deki Reader v100 devices, participated in this evaluation: Diepsloot Clinic, Eyethu Clinic, Hillbrow Clinic, OR Thambo Clinic, Themba Lethu Clinic, Windsor Clinic, Yeoville Clinic and the Dreams Team Mobile HTS clinic. In total, 52 HCWs had training to participate in this evaluation.

Software was upgraded between phase 1 and phase 2 to allow for automated result reporting and algorithm enforcement by the Deki Reader v100, through the use of an onscreen job aid. The HCW was obliged to acknowledge the procedures performed and automated RDT assessment was not possible if the algorithm procedures were not followed. After entering the visual result, the RDT was inserted into the device for image capture and automated assessment. When a screening result was recorded as HIV-positive, the HCW was prompted to continue to confirmatory screening, following the same process. The HCW could not access the automated analysis results to ensure ongoing SOC and the HCW visual result call (gold standard) was used for patient management.

There was a change to the HTS algorithm mid-evaluation when the Gauteng Department of Health RDT supplier was changed as per tender RT41–2017 [33]. The HTS algorithm used from 8 October to mid-December 2017 used the Advanced Quality Anti-HIV (1&2) Test Card (Advanced Quality; Intec, Haicang Xiamen, China) for HIV screening, followed by result confirmation with the ABON™ HIV 1/2/0 Tri-Line Human Immunodeficiency Virus Rapid Test. The HTS algorithm used in Gauteng from December 2017 uses ABON™ HIV 1/2/0 Tri-Line Human Immunodeficiency Virus Rapid Test for screening, followed by confirmation with the First Response® HIV 1–2-0 Card Test (First Response; Premier Medical Corporation Ltd., Daman, India). There were delays implementing the December 2017 algorithm change as clinics used up their existing RDT stocks before commencement of the new (all clinics were using the modified algorithm by February 2018). Furthermore, the manufacturer of the Abon RDT modified the printing of the cartridge in December and the RDT could not be recognised by the device. A single software upgrade to allow for the new cartridge design and the new algorithm was required, and became available on 02 February 2018.

End-user surveys were conducted both pre- and post-implementation to determine the HCWs’ perceptions of the usability and performance of the Deki Reader. To eliminate biases, researchers issued the structured questionnaires to managers who administered them to all the end-users. The end-users were asked to rank the Deki Readers based on the perceived usefulness and ease-of-use during testing using a Likert-scale [34] (quantitative analysis). The questionnaire also included open-ended questions (qualitative analysis). Any obvious spelling errors were corrected during data analysis.

### Continuous quality monitoring

The Fionet™ portal allows for storage of all data related to RDT programme(s) using the Deki Reader. This data can be monitored centrally on a continuous basis. The capabilities of the portal were examined to determine the extent of continuous quality monitoring (audit indicators of test and algorithm quality) available to authorised individuals (e.g. region managers). Both the devices and the web portal are accessed through a registered user name and password based on access authorisation determined at study commencement, ensuring that data security is maintained. Only authorised personnel (Fio technical software developers, clinical and laboratory research scientists, and research program managers) had access to anonymised results.

### Statistical analysis

Data collected through the Fionet™ portal were downloaded as Microsoft® Excel (Microsoft® Corporation, Redmond, WA, USA) files and included study number, date of processing, HCW login, RDT used, visual interpretation and automated interpretation (both including HIV-1 and HIV-2 if included on the RDT). The visual RDT interpretation (SOC) was considered the reference for all analyses. All data was adjudicated by a Fio expert, in order to improve the analysis software. Performance evaluation analyses of the Deki Reader result outputs compared to visual interpretation (considered the reference standard) included concordance, sensitivity and specificity, and inter- and intra-device result reproducibility (only determined in the laboratory setting). Concordance included invalid specimens, but excluded errors and specimens that were not processed, while sensitivity and specificity only included specimens with a valid HIV positive or negative result. Acceptability for reproducibility, concordance, sensitivity and specificity was set at ≥98%. Statistical analyses were performed using Microsoft® Excel 2016, Stata version 12-SE (StataCorp LLC, College Station, TX, USA) and MedCalc (version 19.1.5, MedCalc Software, Ostend, Belgium). The HCW user experience feedback (quantitative and qualitative, as described above applying the Likert score and descriptive experiences) was captured in Microsoft® Excel 2010 tables and positive or negative experiences summarised using a heat map approach.

## Results

### Laboratory evaluation

The laboratory evaluations of both the Deki Reader v100 and the Deki Reader v200 showed that automated RDT analysis was comparable with SOC (visual RDT analysis). Overall precision (Table 1) was 98.3% for both intra- and inter-device precision for the Deki Reader v100, with overall sensitivity of 98.7% (95% CI: 93.2–100) and specificity of 96.2% (95% CI: 80.4–99.9), across 121 RDTs processed and uploaded to Fionet™. A number of results (*n* = 12) were not uploaded (connectivity) and there were two errors recorded. If HCW entry error (*n* = 1), which caused a false positive result when the Deki Reader was compared to SOC, is taken into account, the intra- and inter-device precision improves to 99.2%. The intra- and inter-device precisions were 99.3 and 99.2% respectively for the Deki Reader v200. The Deki Reader v200 intra-device sensitivity was 100% (95% CI: 95.2–100) with specificity of 98.7% (95% CI: 92.8–100), and inter-device sensitivity was 98.5% (95% CI: 92.1–100) with 100% specificity (95% CI: 95.1–100). The overall intra-device (*n* = 150) sensitivity and specificity were 100% (95% CI: 95.2–100) and 98.7% (95% CI: 92.8–100) respectively, while inter-device (*n* = 142) sensitivity and specificity were 98.5% (95% CI: 92.1–100) and 100% (95% CI: 95.1–100). If HCW entry error in the Deki Reader v200 evaluation was addressed, similarly to the Deki Reader v100 evaluation, the intra-device precision improved to 100%. Discordant results (*n* = 1 per evaluation) were linked to the presence of artefact in the region of the RDT HIV-2 test line, which affected the overall HIV status (Table 2). Similar artefact was observed in several specimens (*n* = 33 concordant and *n* = 6 discordant with SOC), but did not affect the overall HIV status.

**Table 1:** Precision of the Deki Reader using laboratory derived specimens

Deki Reader Version	RDT brand	Result compared to SOC					^**b**^Concordance n (%)
		HIV positive		HIV negative		Deki & HCW invalid (true invalid)^**a**^	
		True	False	True	False		
**Intra-instrument reproducibility (5 specimens read 3 times on the same instrument (*n*** **= 3 instruments))**							
**V100 (*n*** **= 45)**	^c^ First Response® HIV-1-2-0	25	^d^ 1	16	–	–	41 (97.6)
	Determine™ HIV-1/2 Ag/Ab Combo	29	–	–	–	16	45 (100)
	^c,g^ Uni-Gold HIV	24	–	9	^d^ 1	–	33 (97.1)
**Overall (*n*** **= 121**^**c,g**^**)**		**78**	**1**	**25**	**1**	**16**	**119 (98.3)**
**Intra-instrument reproducibility (5 specimens read 5 times on the same instrument)**							
**V200 (*n*** **= 50)**	First Response® HIV-1-2-0	25	–	25	–	–	50 (100)
	One Step Anti-HIV (1&2)	25	^e^ 1	24	–	–	49 (98.0)
	HIV 1/2/0 Tri-Line	25	–	25	–	–	50 (100)
**Overall (*n*** **= 150)**		**75**	**1**	**74**	**–**	**–**	**149 (99.3)**
**Inter-instrument reproducibility (5 specimens read across 3 instruments (3 reads per instrument))**							
**V100 (*n*** **= 45)**	^c^ First Response® HIV-1-2-0	25	^d^ 1	16	–	–	41 (97.6)
	Determine™ HIV-1/2 Ag/Ab Combo	29	–	–	–	16	45 (100)
	^c,g^ Uni-Gold HIV	24	–	9	^e^ 1	–	33 (97.1)
**Overall (*n*** **= 121**^**c,g**^**)**		**78**	**1**	**25**	**1**	**16**	**119 (98.3)**
**Inter-instrument reproducibility (10 specimens read across 5 instruments)**							
**V200 (*n*** **= 50)**	^f^ First Response® HIV-1-2-0	21	–	25	–	–	46 (100)
	One Step Anti-HIV (1&2)	25	–	24	^d^ 1	–	49 (98.0)
	^f^ HIV 1/2/0 Tri-Line	21	–	25	–	–	46 (100)
**Overall (*n*** **= 142**^**f**^**)**		**67**	**0**	**74**	**–**	**–**	**141 (99.2)**

^a^Numbers indicate concordant invalid results from both Deki Reader and HCW. No false invalid specimens were observed

^b^Concordance includes invalid specimens, but excludes errors and specimens that were not processed

^c^Specimens not uploaded to Fionet™ (First Response® HIV 1–2-0 *n* = 3; Uni-Gold HIV *n* = 9)

^d^Presence of artefact

^e^HCW transcription error

^f^Specimens not captured by HCW (First Response® HIV-1-2-0 *n* = 4; HIV 1/2/0 Tri-Line *n* = 4)

^g^ Deki Reader error (*n* = 2)

**Table 2:** HIV-1 and HIV-2 results (RDTs with separate target lines for HIV-1 and HIV-2)

Deki Reader Version	RDT brand	HIV-1 Result					HIV-2 Result				
		Positive		Negative		^a^Concordance n (%)	Positive		Negative		^**a**^ Concordance n (%)
		True	False	True	False		True	False	True	False	
**Intra-instrument reproducibility (5 specimens read 3 times on the same instrument (*n*** **= 3 instruments))**											
^**b**^**V100** **(*n*** **= 42)**	First Response® HIV-1-2-0	12	0	30	0	42 (100)	13	^d^ 1	28	–	41 (97.6)
**Intra-instrument reproducibility (5 specimens read 5 times on the same instrument)**											
**V200** **(*n*** **= 50)**	First Response® HIV-1-2-0	25	0	25	0	50 (100)	0	^d^ 5	45	0	45 (90.0)
	HIV 1/2/0 Tri-Line	25	0	25	0	50 (100)	^e^ 10	0	40	0	50 (100)
**Inter-instrument reproducibility (5 specimens read across 3 instruments (3 reads per instrument))**											
^**b**^**V100 (*n*** **= 42)**	First Response® HIV-1-2-0	12	0	30	0	42 (100)	13	^d^ 1	28	0	41 (97.6)
**Inter-instrument reproducibility (10 specimens read across 5 instruments)**											
^**c**^**V200** **(*n*** **= 46)**	First Response® HIV-1-2-0	21	0	25	0	46 (100)	^e^ 4	^d^ 1	41	0	45 (97.8)
	HIV 1/2/0 Tri-Line	21	0	25	0	46 (100)	^e^ 6	0	40	0	46 (100)
**Laboratory-derived specimen concordance (*n*** **= 158)**											
**V200** **(*n*** **= 158)**	First Response® HIV-1-2-0	110	^f,g^ 5	43	0	153 (96.8)	13	^g^ 6	137	^f^ 2	150 (94.9)
	HIV 1/2/0 Tri-Line	112	^f,g^ 7	39	0	151 (95.6)	45	^g^ 5	101	^f^ 7	146 (92.4)

^a^Concordance includes invalid specimens, but excludes errors and specimens that were not processed

^b^Specimens not uploaded to Fionet™ (*n* = 3)

^c^Specimens not captured (*n* = 4)

^d^Presence of artefact: discordant HIV-2 positive results affecting overall HIV result

^e^Presence of cross reactivity: concordant HIV-2 positive results (this was linked to two specimens)

^f^HCW transcription error

^g^Deki Reader error

The concordance analysis of laboratory specimens (Table 3) was performed on 774 different specimens separated across different RDTs for the Deki Reader v100 evaluation. Acceptable overall concordance between the Deki Reader v100 and SOC results was observed both before (98.1%) and after (99.5%) data adjudication, and across all RDTs analysed: First Response® HIV 1–2-0: 100%, Alere Determine™ HIV-1/2 Ag/Ab Combo: 98.8% and Uni-Gold™ HIV: 99.6%. Overall sensitivity and specificity for the v100 were 99.5% (95% CI: 98.2–99.9) and 99.4% (95% CI: 97.9–99.9) respectively, with the sensitivity and specificity of individual RDTs also acceptable. Two positive specimens by SOC (Uni-Gold HIV) were recorded as errors by the Deki Reader v100; in both cases the error was caused by invalid control line. Four specimens were misclassified (2/341 negative specimens and 2/397 positive specimens). The two false negative results (Alere Determine™ HIV 1/2 Ag/Ab Combo RDT (*n* = 1) and Uni-Gold™ HIV RDT (*n* = 1)) were caused by target capture lines with a width < 30% of the cassette window; the standard Deki Reader configuration is that a line is not present if its width is < 50% of the window. The two false positive results (both Alere Determine™ HIV 1/2 Ag/Ab Combo RDT) were the direct result of air bubbles in the membrane at the time of analysis (15 min), which were misinterpreted as test lines. All errors and misclassifications were related to the individual RDT used, rather than to the performance of the Deki Reader v100.

**Table 3:** Laboratory evaluation of the Deki Reader comparing device versus SOC results

Deki Reader Version	RDT brand	Result compared to SOC								^**a**^Concordance n (%)
		HIV positive		HIV negative		^**a**^Sensitivity % (95%CI)	^**a**^Specificity % (95%CI)	Deki & HCW invalid (true)	Deki invalid (false)	
		True	False	True	False					
^**b**^**V100**	First Response® HIV-1-2-0 (*n* = 253)	128	–	118	–	100 (97.2–100)	100 (96.9–100)	7	–	253 (100)
	Determine™ HIV-1/2 Ag/Ab Combo (*n* = 259)	135	^c^ 2	94	^d^ 1	99.3 (96.0–100)	97.9 (92.7–99.8)	27	–	256 (98.8)
	^e^ Uni-Gold HIV (*n* = 262)	132	–	127	^d^ 1	99.3 (95.9–100)	100 (97.1–100)	–	–	259 (99.6)
	**Overall (*n*** **= 772)**	**395**	**2**	**339**	**2**	**99.5** **(98.2–99.9)**	**99.4** **(97.9–99.9)**	**34**	**–**	**768 (99.5)**
**V200**	First Response® HIV-1-2-0 (*n* = 158)	113	2	43	–	100 (96.8–100)	95.6 (84.9–99.5)	–	–	156 (98.7)
	One Step Anti-HIV (1&2) (*n* = 158)	109	2	40	–	100 (96.7–100)	95.2 (83.8–99.4)	1	^f^ 6	150 (94.9)
	HIV 1/2/0 Tri-Line (*n* = 158)	114	5	39	–	100 (96.8–100)	88.6 (75.4–96.2)	–	–	153 (96.8)
	**Overall (*n*** **= 474)**	**336**	**9**	**122**	**–**	**100** **(98.9–100)**	**93.1** **(87.4–96.8)**	**1**	**6**	**459 (96.8)**

^a^Concordance includes invalid specimens, but excludes errors and specimens that were not processed. Sensitivity and specificity only includes specimens that gave a valid positive or negative result

^b^Deki Reader data was adjudicated by Fio as part of the process to optimise the output data (software optimisation)

^c^Air bubble over control line, linked to RDT

^d^Line < 50% of width, software adjustments required

^e^Deki Reader v100 error (*n* = 2)

^f^Deki Reader v200 invalid control line

The Deki Reader v200 concordance (Table 3) was evaluated using 158 specimens processed on three different RDTs. Concordance in comparison to SOC was lower than expected (96.8%), with nine (1.9%) false positive results recorded, and only the First Response® HIV 1–2-0 displaying concordance above the acceptable limit (98.7%). The One Step Anti-HIV (1&2) concordance was 94.9%, primarily due to the Deki Reader v200 reporting invalid control line position (*n* = 6). When this data was adjudicated (increased flexibility in the location of the target line within the RDT), the One Step Anti-HIV (1&2) concordance improved to 98.6% and overall concordance improved to 98.0% (acceptable). The HIV 1/2/0 Tri-Line concordance was 96.8%, with five false positive classifications by the Deki Reader v200; this may indicate that refinement of the new software is required (device learning). HCW entry errors (First Response® HIV 1–2-0: *n* = 4; HIV 1/2/0 Tri-Line: *n* = 6) were observed when HIV-1 and HIV-2 results were entered in reverse (Table 2). While sensitivity was excellent (100% (95% CI: 98.9–100)), specificity was lower than acceptable at 93.1% (95% CI: 87.4–96.8), with the HIV 1/2/0 TriLine RDT specificity less than 90% (88.6% (95% CI: 75.4–96.2)).

### Field evaluation

Nineteen (82.3%) trained HCW participated in the first field evaluation, with 3159 RDT results successfully uploaded to Fionet™. On average 527 results were uploaded per month, but with a wide range (153, 1573), and the majority were uploaded during July 2017. The average number of records uploaded in <10 min was 95.8% (range: 94.2, 98.0), and all records were uploaded within a maximum of seven days. The quality data transmission (QDT, 10% of records) was consistently 100%. No battery failures were experienced over the course of the study and 90% (*n* = 18) of Deki Reader v100 devices showed no decrease in functionality, However, 10% (*n* = 2) devices, both placed with mobile units, failed to function optimally towards the end of the evaluation.

Thirty-six (69.2%) of HCW trained participated in the second field trial (concordance evaluation), although thirteen HCW processed less than ten specimens. Four HCW processed more than 400 specimens, ten processed between 100 and 300 specimens, and a further nine HCW processed between 50 and 100 specimens. A total of 4460 patients were enrolled in this field evaluation, 2463 (55.2%) under Algorithm 1 and 1997 (44.8%) under Algorithm 2. After data adjudication, concordances were 99.3 and 96.5% (screening), and 99.7 and 100% (confirmatory) for Algorithms 1 and 2 respectively (Table 4). Overall sensitivity and specificity across all specimens were 99.8% (95% CI: 99.2–100) and 97.7% (95% CI: 97.2–98.2) respectively, with decreased Deki Reader specificity observed for the HIV 1/2/0 TriLine RDT which was common to both algorithms (93.8% as the confirmatory RDT in Algorithm 1 and 96.1% as the screening RDT in Algorithm 2).

**Table 4:** Clinical evaluation of the Deki Reader v100

RDT brand	Result compared to SOC							^**a**^Concordance n (%)	Error/not analysed (Deki)
	HIV positive		HIV negative		^**a**^Sensitivity % (95%CI)	^**a**^Specificity % (95%CI)	Deki & HCW invalid (true)		
	True	False	True	False					
**Algorithm 1: Screening: Advanced Quality Anti-HIV (1&2); Confirmatory: HIV 1/2/0 Tri-Line**									
Advanced Quality Anti-HIV (1&2) (*n* = 2463)	386	15	1817	2	99.5 (98.2–99.9)	99.2 (98.7–99.5)	32	2252 (99.3)	196
HIV 1/2/0 Tri-Line (*n* = 352)	261	^b^ 1	15	–	100 (98.6–99.8)	93.8 69.8–99.8)	5	281 (99.7)	70
**Algorithm 2: Screening: HIV 1/2/0 Tri-Line Confirmatory: First Response® HIV-1-2-0**									
HIV 1/2/0 Tri-Line (*n* = 1997)	153	64	1588	–	100 (97.6–100)	96.1 (95.1–97.0)	-	1741 (96.5)	192
First Response® HIV 1–2-0 (*n* = 129)	115	–	1	–	100 (96.8–100)	100 (2.5–100)	1	117 (100)	12
**Overall RDTs (*n*** **= 4941)**	**915**	**80**	**3421**	**2**	**99.8** **(99.2–100)**	**97.7** **(97.2–98.2)**	**38**	**4374 (97.8)**	**470**

^a^Concordance includes invalid specimens, but excludes errors and specimens that were not analysed. Sensitivity and specificity only includes specimens that gave a valid positive or negative result

^b^SOC positive and Deki Reader invalid. Specimen was incorrectly processed and reported; this a HCW error. See Fig. 3c

**Fig. 3:**
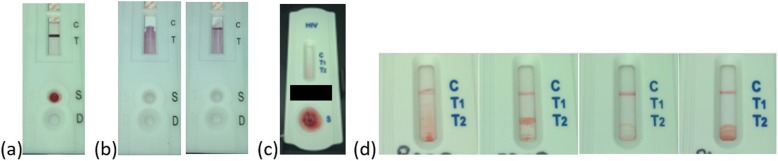
Selected RDTs images linked to discordant results (Field evaluation phase 2). Discordant results (between visual and automated RDT analysis) were further examined, using the images captured by the Deki Reader and transmitted to the Fionet portal. Specific examples, as discussed in the text, are shown. **a** Advanced Quality Anti-HIV (1&2) RDT with misaligned membrane. **b** Advanced Quality Anti-HIV (1&2) RDTs with insufficient movement of specimen through the membrane. **c** HIV 1/2/0 Tri-Line confirmatory RDT misanalysed by the HCW (positive call); specimen did not travel through the membrane. **d** Examples of HIV 1/2/0 Tri-Line screening RDT membranes with artefact

**Fig. 4:**
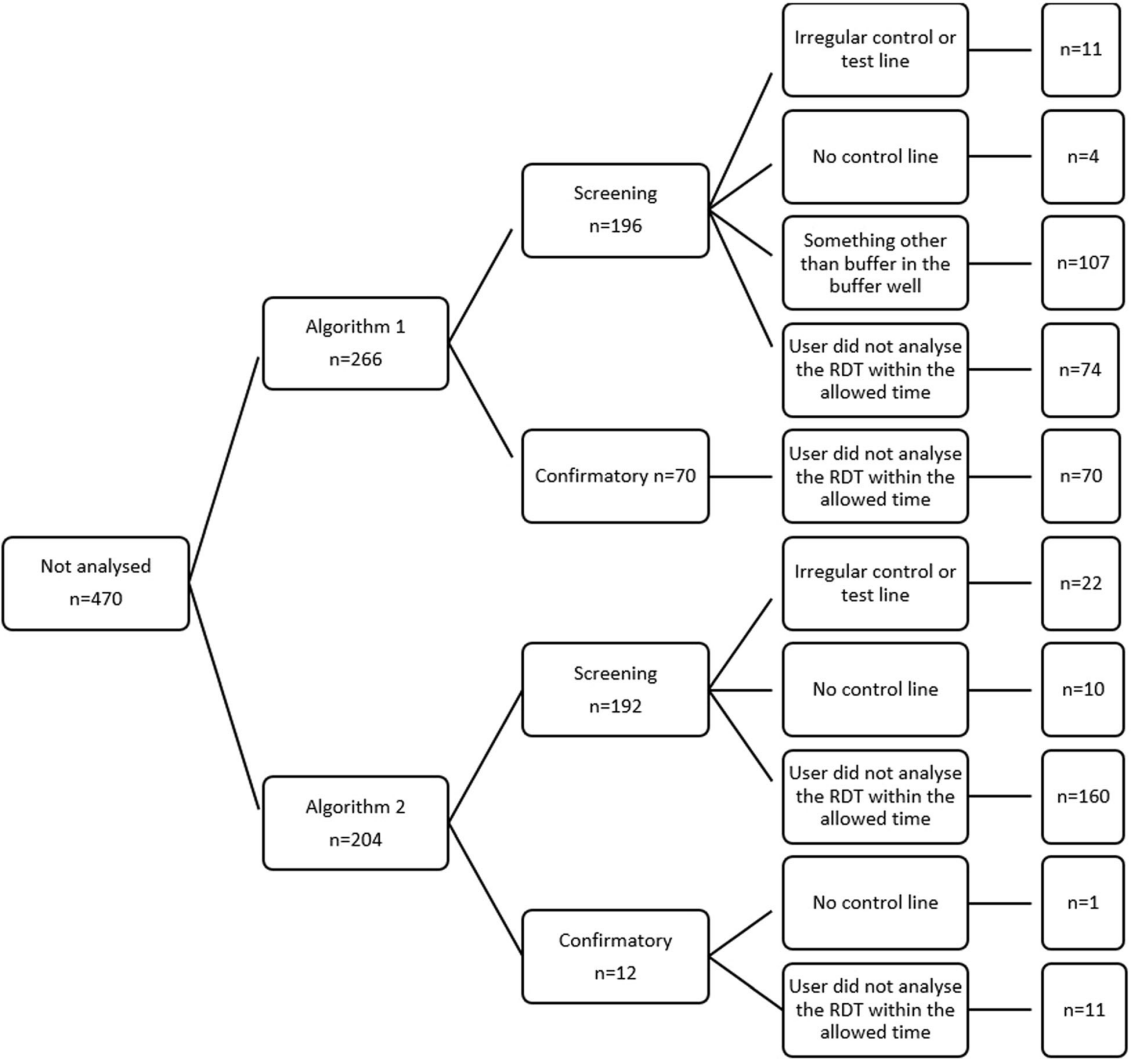
Summary of RDTs (*n* = 470) unable to be analysed by the Deki Reader v100 during the clinical concordance field evaluation. In 10.5% of RDTs, the Deki Reader gave an error flag (unable to be analysed) rather than a result. These flags can be used for continuous quality monitoring of HCW performance and RDT quality. Under Algorithm 1 there were 266 specimens that were not analysed, of which 144 were not analysed within the required time period (HCW error). A high number (*n* = 107) of the Advanced Quality™ Rapid HIV (1&2) RDT were not analysed as the blood was added to the buffer well (HCW error). The remaining errors were found on the RDT (4 with no control line and 11 with irregular test or control lines). Similarly, under Algorithm 2 (*n* = 204), 171 flags indicated that the RDT had not been analysed within the required time period (HCW error*), with a slightly higher number of RDT errors (11 with no control line and 22 with irregular control or test lines). *While these errors are linked to HCW performance, in this case it was determined that the majority of these errors occurred on one Deki Reader, where the device drawer did not detect closure, leading to the allowable timeframe expiring; once the drawer was fixed, this error was not noted again

There were a number (1.4%, *n* = 32) of invalid (no result by both SOC and automated analysis) Advanced Quality Anti-HIV (1&2) RDTs in Algorithm 1. Furthermore, 15 false positive screening RDTs were recorded, of which twelve were true negative results and three should have been repeated: in one case the RDT strip had shifted (Fig. 3a) and in the remaining two cases the specimen did not move completely through the RDT membrane; it was proposed that insufficient buffer was added to the RDT, which could indicate HCW retraining is required (Fig. 3b). One false positive confirmatory RDT compared to SOC was recorded and could be linked to HCW analysis error, with the specimen not moving through the RDT membrane (Fig. 3c). A high number of false positive (3.6%) HIV 1/2/0 Tri-Line RDTs were recorded under algorithm 2; 53/64 were linked to high levels of artefact in the region of the HIV-2 target line (Fig. 3d), which skewed the automated analysis, while the remaining 11 were true negative results. Specimens which were not analysed by the Deki Reader v100 are summarised in Fig. 4. The majority of errors were linked to the RDT not being analysed within the allowed timeframe (*n* = 315, 67%) and to blood being added to the buffer well (*n* = 107, 22.8%), which was only observed with the Advanced Quality Anti-HIV (1&2) RDT. Both of these are HCW errors, although the former was reported by HCW to be caused by the Deki Reader v100 RDT drawer not detecting closure in multiple instances.

**Fig. 5:**
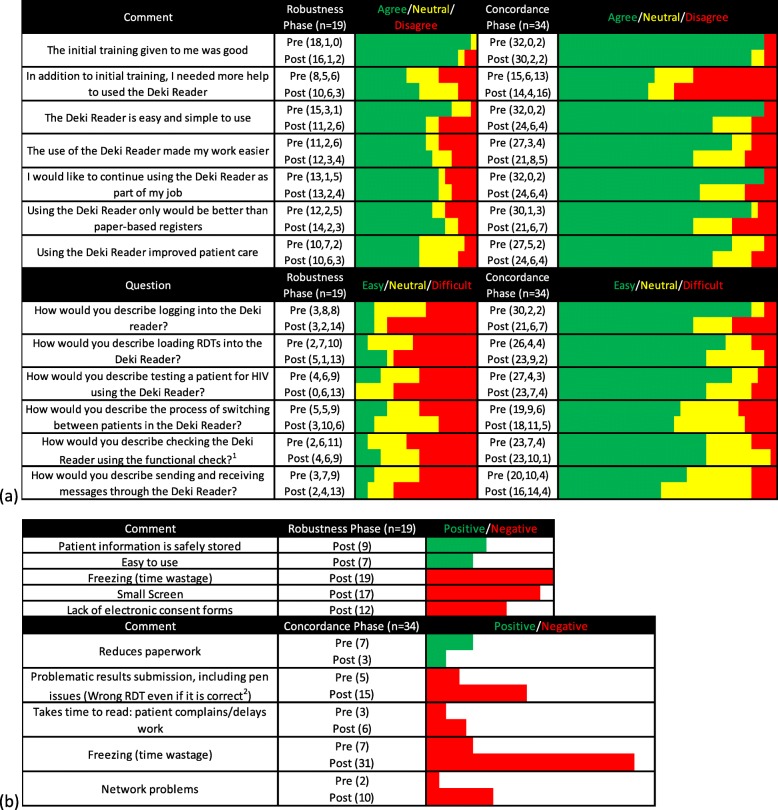
Summary of HCW feedback (pre- and post- field evaluation). HCWs completed feedback forms after Deki Reader v100 training (pre-evaluation) and post-field evaluation for both the robustness (*n* = 19) and concordance (*n* = 34) evaluations. The feedback results are summarised as a heat map: green = positive response, yellow = neutral response, red = negative response. The forms consisted of defined comments/questions and free text provided by the HCWs. **a** The defined comments/questions used a Likert scale and the HCW were asked to agree (positive response) or disagree (negative response), or to define as easy (positive response) or difficult (negative response), with a neutral option also available. There was more HCW ambivalence over the use of the technology post the field evaluation (increased red/yellow). **b** The most frequently recorded comments are shown, and coded as positive (green) or negative (red) responses out of the total number of HCW per phase. No comments were provided for the pre-evaluation questionnaire in the robustness phase. Notes: ^1^The functional check was a manufacturer designed RDT to be used to ensure that the instrument was functioning correctly at the start of each day. ^2^The pens used to write patient initials on the RDT absorbed into the RDT cassette plastic between the initial RDT image capture and the result capture (after processing time), causing the Deki Reader v100 to reject the RDT as a different specimen

HCW surveys were completed post-training and post-field evaluation, with 19 HCW (100%) participating in the first field evaluation feedback and 34 HCW (65.4%) participating in the second field evaluation feedback (Fig. 5). Overall, HCWs were more ambivalent about using the device after several months of use in the field, with many indicating that it slowed the clinic workflow. The most frequent end-user complaint (100 and 91.2% from the first and second field evaluations respectively) was instrument suspension (‘freezing’). The size of the screen, even when using stylus pens, and the lack of electronic complaint forms were common complaints during the first field evaluation. Two device manufacture issues reported during the second field evaluation were both linked to problematic result capture by the Deki Reader v100: (1) the RDT drawer did not detect closure immediately and the result was then not captured as processing time was exceeded and (2) the marker pens used to write patient initials on the RDT absorbed into the RDT cassette plastic between the initial RDT image capture and the result capture (after processing time), causing the Deki Reader v100 to reject the RDT as a different specimen. HCW at high volume testing sites also stated that the use of the Deki reader slowed the workflow and this led to patient complaints. Based on HCW feedback, Fio Corporation redesigned the Deki Reader with an improved camera, a larger screen and increased stability, and also removed the need for third party part supply. The Deki Reader v200 showed good robustness and battery life in the laboratory, but evaluation of device robustness in the field is ongoing.

### Continuous quality monitoring

The Fionet™ portal enabled centralised monitoring of the devices, and also of individual HCW and clinics, which can support early detection of breaches in protocol. For example, in the case of the RDT drawer not closing, two HCW using the same instrument (consequtively and at different sites) were responsible 90.0 and 89.5% of these errors, which comprised 30% of time expired errors. Once the drawer was replaced both HCW were not linked to similar error issues. This type of incident can be addressed timeously to ensure continued quality at all testing sites, through assessment of HCW, devices and clinic procedures. Weekly encounters at clinics are tracked, as are error rates across all sites. Lastly, the actual RDTs can be monitored for lot errors; on the recommendation of the field trial managers, lot numbers can now be recorded on the devices.

### Target product profile

A TPP was designed based on the existing TPP for viral load monitoring in resource limited settings [34] and on knowledge gained through experience in the field, in consultation with Deki Reader training staff. The Deki Reader v100 (laboratory and field evaluations) and Deki Reader v200 (laboratory evaluation) showed acceptable performance, meeting either minimal or optimal criteria throughout. Key criteria met, beyond sensitivity and specificity, include: (1) the ability to evaluate and capture RDTs for different diseases and from different suppliers (although ‘software training’ is required); (2) connectivity to a central database (in this case www.fionet.com) through a local mobile data service provider; (3) a robust, electricity-independent system with rechargeable batteries; and (4) the ability to log multiple patients concurrently, yet ensure that the correct RDT is analysed for each patient.

## Discussion

The HTS programme in South Africa has been expanding since 2000 [35] and RDT use is significant. Notable landmark steps include the 2010 HTS guideline amendments to allow non-healthcare workers to test for HIV using RDTs [36], the launch of nurse-initiated ART [37] and, most recently, UTT [9] and self-testing guidelines [38]. Quality HIV screening has always been important to patient well-being and highlights the need for correctly administered and evaluated RDTs [1, 39]. Next steps should thus include improved quality monitoring, improved surveillance to enable program optimisation and improved management of patient linkage to care. Devices that are able to monitor the testing process, automatically evaluate the RDT and upload data to a centralised system can address these gaps and potentially standardise the quality of care across a variety of settings, ranging from true remote POC through to urban clinics. A central database allows all sites and RDT testers to be monitored in real-time, meaning that anomalies in the system, such as RDT batch errors and decreased staff proficiency, can be detected early [24]. The readily available real-time data allows for effective monitoring and surveillance of the program and remote management of HCWs, including targeted identification for retraining, which was one of the areas identified by Johnson et al. (2017) [1], and the centralised storage of countrywide HIV screening results allows data to be instantly available for HIV prevalence monitoring. Although this study focused on feasibility and RDT reader performance, the quality indicators observed did indicate gaps in the field, which are not otherwise recorded or monitored. The centralisation of field data can potentially enable managerial oversight of clinics, HCW and RDTs in the field for follow-up, corrective action and quality monitoring of the HTS programme, as well as for optimised HIV surveillance.

This study included a number of evaluations. Sensitivity and specificity between the visual and automated RDT analysis showed variability, while concordance was consistently acceptable. The laboratory evaluations of both the Deki Reader v100 and the updated Deki Reader v200, showed intra- and inter-device precision of > 99%. Similarly, concordance was consistently above 95% (minimal criterion) and, excluding HCW errors and artefact, close to 99%. Concordance in the field was also high, 99.3 and 99.7% for screening and concordance respectively for Algorithm 1, and 96.5% (screening) and 100% (concordance) for Algorithm 2. The decreased screening concordance under Algorithm 2 was primarily caused by high levels of artefact present in the region of the HIV-2 target line on the HIV 1/2/0 Tri-Line RDT, which skewed the automated analysis. Options to overcome this are being investigated further by Fio Corporation, and the addition of lot number recording to the software was recommended to determine whether specific RDT batches were affected. The artefact could also be linked to imperfect processing, which would indicate a need for HCW retraining; review of RDTs centrally (as is not currently possible) would allow this to be detected rapidly. The processing error rate (9.5%) was slightly higher than that reported by Kalinga and colleagues (2018) for malaria RDTs [24], but was notably linked to the RDT not being assessed in the allowable time period. This did highlight HCW retraining, but was also found to relate to a drawer not closing; once the drawer was replaced, this error decreased. These, and examples described in the results section, show how the Fionet™ portal, when optimally monitored, can trigger interventions remotely to allow rapid corrective action in the field, be the errors linked to HCW, RDTs or to the Deki Reader itself. Recent evaluation of the Deki Reader v100 to monitor malaria RDTs showed a 97% decrease in processing errors when using the device and applying quality monitoring through the Fionet™ portal [24]. Identification of these audit indicators (and their rate of monitoring: daily, weekly, monthly) and the responsibility of monitoring and reporting need to be defined within the laboratory and clinical interface ecosystem and responsibilities assigned, especially in large HIV programs.

In the field, the overall robustness, quality data capturing and connectivity, and record upload speed were good. The Deki Reader was able to capture and transmit reliable data for monitoring and evaluation purposes (100%), and to store patient results until data upload if no connectivity was available. Neither of these was previously widely possible when using RDTs for HIV diagnosis, with conventional data records being paper-based, although certain mobile phone-based applications have been suggested [40]. The reliability (100%) of the batteries show the potential of the Deki Reader as a tool for POC testing or outreach testing in rural areas, where limited access to electricity may be a problematic factor, provided that it is possible to recharge the device every two to three days. While the Deki Reader v100 was robust, the failure of two instruments (10%) in the mobile testing centres towards the end of the evaluation indicate a need for further assessment in mobile settings. Furthermore, it is concerning that all end-users reported system freezes. Suggestions for this were the use of third-party software, possibly intermittently clashing with the Deki Reader operating system, or a lack of random access memory and/or storage space. The Fio Corporation addressed these concerns by bringing the technology manufacture in-house with the Deki Reader v200, and ongoing field evaluations will determine if this problem has been corrected, which is vital before widespread rollout of the devices is feasible.

While the HCW were initially positive regarding the Deki Reader v100 improving their workflow, the end-user acceptance of the Deki Reader showed more ambivalence or dislike of the device in the post-evaluation surveys. The primary reasons for this were the system freezes, which have been addressed and will be re-examined during the Deki Reader v200 field evaluation, and that the Deki Reader will not allow a strip to be analysed prior to completion of the incubation period. While the extended analysis time does “irritate the patient as they don’t want to wait”, ensuring proper adherence to protocols does ensure the provision of high quality HTS services. A recent study showed that many HCW read the results too early and misdiagnose patients [12], in addition to the subjective nature of visual RDT interpretation [24]. Patient acceptance of the device should also be formally analysed in future studies, as comments from patients were received via the HCW, indicating that patients did not like the time taken to log data (particularly with system freezes) or that they had to wait the recommended length of time before results were shared.

It is proposed that the Deki Reader may also enhance HTS tester performance through the on-board systematic guidance and quality control checks. The use of both the paper-based records and the Deki Reader v100 in the field evaluation did increase the HCW time burden, which may have influenced their opinions, and ideally paper-based registers will be discontinued if automated, connected RDT analysers are implemented. However, the registers were useful in the data analysis, as both field and laboratory staff reported that if the incorrect visual result was entered onto the Deki Reader (both versions), there was no option to correct it. When comparing the paper-based results to the RDT images and entered visual results, it was noted that the majority of discordant specimens (Deki Reader compared to SOC) were not truly discordant. Based on this, the software is being modified to allow result correction (visual) prior to automated analysis reporting. The HCW will also have the option to dispute the automated result, such as in the case of artefact, although this is still under development.

The Deki Reader v100 met the majority of criteria suggested for the TPP and the Deki Reader v200 will be similarly assessed in the field. The robustness of the device should be evaluated further in more stringent environments, including increased humidity and more extreme temperatures, and full costing analyses must be performed. Although not part of the TPP, it should be mentioned that the value of a fixed, rather than mobile, device is that the distance between the device camera and RDT, remains constant irrespective of the RDT. The closed chamber further standardises the light environment when an image is captured, irrespective of the surrounding environmental light conditions. The presence of artefact is being addressed, as this was the most worrying TPP criterion not met, albeit only with one RDT brand. However, ongoing software optimisation and HCW training, including repeating RDTs when artefact is visible, may address this issue.

It was noted that a potential limitation of the technology is that the introduction of any new RDT requires software modification, which requires time and validation prior to implementation. In addition, the RDT reader developer will require access to the new RDT to be used in the field and implementation partners need to be aware of time delays for technology optimisation. However, devices not locked to specific RDTs are important when using the device in different settings, with varying algorithms, RDT types and HTS protocols being required dependant on country needs. The ability of the Deki Reader to accept new software (pushed through to the device from a central point) and the flexibility of the software, as shown during the algorithm change mid field evaluation, enables the device to adapt to the needs of the settings and allows the RDT market to remain competitive.

Lastly, with the increased availability of HIV self-testing, which is performed by the untrained layman, and the need for linkage to care for persons with a positive result [41, 42], the Deki Reader may also have a role beyond HTS clinics. An investigation into the acceptability of siting Deki Readers in private areas at selected pharmacies or similar for HIV self-testing could ensure better quality results and the option for results to be sent to a health-care provider. The device is simple to use and may be an alternative to downloading smartphone applications to submit self-processed RDTs for analysis. At a minimum, the delinked information could be available for surveillance purposes. Expanding the Fionet™ portal capability to incorporate geographic information systems mapping could be further valuable tool for program surveillance.

## Conclusion

Contemporary technologies have the potential to enhance the provision of healthcare services, particularly at POC. The Deki Reader provides a means for continuous quality control and reliable quality assessment in rapid diagnosis of HIV using RDTs. This includes effective and efficient patient care and data capturing, as well as high connectivity capabilities, and the device has the potential to be deployed to rural areas of South Africa to optimise HTS coverage. The technology can also be deployed for HIV RDT monitoring in settings where the Deki Reader v100 already has a large footprint in the diagnosis of febrile malaria. However, ongoing and agile measures should be in place to mitigate against all end-user challenges to ensure continuous and consistent acceptability of the device throughout the HIV value chain.

## Data Availability

The datasets generated and/or analysed during the current study are not publicly available due to patient confidentiality, but are available from the corresponding author on reasonable request.

## Abbreviations

ART: Antiretroviral therapy
HCW: Health care worker
HIV: Human Immunodeficiency Virus
HTS: HIV testing services
NHLS: National Health Laboratory Service
PLWH: People living with HIV
POC: Point of care
QDT: Quality data transmission
RDT: Rapid diagnostic test
SEAD: Strategic Evaluation, Advisory & Development
SOC: Standard of care
TPP: Target product profile
UTT: Universal test and treat
WHO: World Health Organisation
